# Functional Connectivity During Handgrip Motor Fatigue in Older Adults Is Obesity and Sex-Specific

**DOI:** 10.3389/fnhum.2018.00455

**Published:** 2018-11-13

**Authors:** Joohyun Rhee, Ranjana K. Mehta

**Affiliations:** ^1^Department of Occupational and Environmental Health, School of Public Health, Texas A&M University, College Station, TX, United States; ^2^Department of Industrial and Systems Engineering, Texas A&M University, College Station, TX, United States

**Keywords:** BMI, gender, fatigability, brain activation and networks, fNIRS

## Abstract

The prevalence of obesity in older adults, particularly in females, is increasing rapidly and is associated with declines in both the brain and physical health. Both the obese and the female populations have shown greater motor fatigue than their counterparts, however, the central neural mechanisms for fatigue are unclear. The present study measured fatigue-related functional connectivity across frontal and sensorimotor areas using functional near-infrared spectroscopy (fNIRS). Fifty-nine older adults (30 non-obese and 29 obese) performed submaximal handgrip motor fatigue until voluntary exhaustion. Functional connectivity and cerebral hemodynamics were compared across eight cortical areas during motor fatigue and across obesity and sex groups along with neuromuscular fatigue outcomes (i.e., endurance time, strength loss, and force steadiness). Both obesity- and sex-specific functional architecture and mean activation differences during motor fatigue in older adults were observed, which were accompanied by fatigue-related changes in variability of force steadiness that differed between groups. While primary indicators of fatigue, i.e., endurance and strength loss, did not differ between groups, the motor steadiness changes indicated different neural adaptation strategies between the groups. These findings indicate that obesity and sex differences exist in brain function in older adults, which may affect performance during motor fatigue.

## Introduction

Obesity is a global public health epidemic (Caballero, 2007). Obesity and its associated health issues place an adverse economic burden on the U.S. health care system, with medical care costs estimated at $147 Billion in 2008 (Finkelstein et al., 2009). Along with comorbidities such as diabetes, hypertension, and sleep apnea, obesity is accompanied with motor impairments, such reductions in muscle power (Lafortuna et al., 2005), increases in motor fatigue (Mehta, 2015), and impaired motor control (Gentier et al., 2013) that may accelerate declines in physical functioning with aging (Lang et al., 2007). These obesity-related decrements in motor skills and functional activity have also been associated with increasing morbidity and mortality in older adults (Aune et al., 2016). This is a critical concern, since ∼40% of adults, particularly women, over the age of 65 are obese (Laura Segal and Martin, 2016). However, mechanisms of motor impairments have largely focused on healthy older adults.

In general, older adults experience declines in both their motor and cognitive capabilities due to the normal age-related degradation of the central nervous system (CNS) and peripheral musculoskeletal system (Frontera et al., 1991; Hughes et al., 1999; Enoka et al., 2003). Specific to the CNS, aging is associated with structural and functional changes in the brain. Ge et al. (2002) reported significantly decreased gray matter volume in older adults compared with younger adults, and the change of cortical volume at the prefrontal area was relatively greater than other brain regions (Good et al., 2001; Jernigan et al., 2001). Gray matter volume is known to be associated with motor performance (Rodrigue et al., 2005), and this relationship may explain age-related motor performance declines. White matter volume also decreases with aging (Jernigan et al., 2001; Ge et al., 2002). Corpus callosum, which is a large bundle of white matter fibers that handles communication between the two hemispheres, is known to be responsible for bimanual coordination and ipsilateral inhibition during unimanual motor tasks (De Gennaro et al., 2004). Therefore, a decrease of white matter volume with aging may affect the stability of motor performance such as balance, coordinative motions, and force control tasks (Sullivan et al., 2010).

Among many age-related changes, fat mass is known to increase with aging and reaches its maximum value after the sixties (Rissanen et al., 1988; Ding et al., 2007). In addition, muscle mass decreases approximately 1% per year and as a result, muscular strength and power also gradually decreases with aging after midlife and can be exacerbated with increased fat mass (Bassey, 1998; Frontera et al., 2000). Older adults who are obese are more likely to experience musculoskeletal injuries and falls (Madigan et al., 2014) and exhibit greater motor fatigue (Maffiuletti et al., 2007; Duan et al., 2018). These impairments have been attributed largely to obesity-specific alterations in peripheral muscular properties, such as changes in muscle mass, fiber type distribution, and contractile properties (Krotkiewski et al., 1990; Hickey et al., 1995; Kriketos et al., 1997; Kern et al., 1999; Newcomer et al., 2001; Tanner et al., 2002). However, whether obesity alters central neural mechanisms of motor fatigue in older adults has not been adequately explored. Additionally, it is important to determine whether there are sex differences in mechanisms for the motor function declines with obesity, because females are more susceptible functional declines (Skelton et al., 1994), particularly with obesity (Jensen and Friedmann, 2002).

Obesity causes structural and functional changes in the brain such as decreased gray matter volume, decreased cortical thickness, and reduced metabolism in the frontal area (Pannacciulli et al., 2006; Willeumier et al., 2011; Marqués-Iturria et al., 2014). Jagust et al. (2005) reported that both central obesity (waist-hip ratio) and aging are negatively correlated with the hippocampal volume that is responsible for information processing and memory consolidation, and positively correlated with hyperintensity lesion of white matter. These obesity-related changes in the brain structures are likely to affect brain function, however, our knowledge on whether functional brain activity is impacted with obesity is limited to cognitive tasks (Gonzales et al., 2014). Only two studies have examined brain function during motor tasks in non-obese and obese adults. Obesity-related decrement in motor performance and concomitant disengagement of the prefrontal lobe during a static upper limb force control task in young adults was reported in (Mehta and Shortz, 2014), while the same group found increased PFC activity during complex and precision gait tasks in older obese adults (Osofundiya et al., 2016) that was associated with similar gait performance when compared to non-obese counterparts. Additionally, other studies that utilized electrical muscle stimulation to understand central vs. peripheral pathways of fatigue attributed obesity-related increased lower limb fatigability in young adults to an increase in central activation failure (Maffiuletti et al., 2007; Pajoutan et al., 2016). However, studies that examine obesity-specific changes in brain function during motor fatigue are limited.

Several imaging studies have reported sex differences in brain structure. For example, males are reported to have greater gray matter (Good et al., 2001) and total brain volume (Sacher et al., 2013) than females after correcting for body size. However, cortical thickness has shown to be greater for females at the frontal, temporal, and occipital areas (Im et al., 2006; Lv et al., 2010). Similarly, structural connectivity of the brain also differs in males and females. Ingalhalikar et al. (2014) reported that females exhibit stronger anatomical connections between the two hemispheres whereas males exhibit stronger connections between the areas within each hemisphere, however, this relationship was shown to be reversed in the cerebellum. These sex differences in the brain structure may explain differences in motor function between males and females. For instance, compared to males, females exhibit greater activation of ipsilateral and bilateral cortical brain regions but lower subcortical activation of the basal ganglia region during finger tapping and motor preparation tasks of similar performance (Lissek et al., 2007). However, whether brain activation differs between males and females during motor fatigue remains unexplored (Hunter, 2014).

When interpreting brain function changes, it is important to understand that while each part of the brain has an assigned role for a certain task, multiple areas of the brain work together to achieve task performance, particularly if the task is performed for a long duration. Therefore, understanding concurrent activation patterns between multiple areas of the brain; also referred to as functional connectivity, is as important as understanding activation of each area in response to the task (Hutchison et al., 2013). The location of functionally connected brain areas can be adjacent to each other, however, it is not necessary for functionally connected areas to be anatomically connected (Bressler and Tognoli, 2006). Determining functional connectivity during motor fatigue may thus help us examine the functions of the brain as a network, thereby contributing toward a better understanding of the mechanisms of motor impairments with obesity and aging, and to explore if these mechanisms differ by sex.

Studies investigating functional connectivity with older and obese adults, and those comparing differences between males and females, have been published. For example, Tomasi and Volkow (2012) reported sex differences in lateralization patterns; females exhibited greater long-range connectivity between hemispheres while males show predominant short-range connectivity within the hemisphere. Goldstone et al. (2016) reported re-organization of the resting state network in older adults. King et al. (2017) argued that age-related declined motor performance is related to overall higher connectivity across networks involved in the large-scale resting state network in older adults when compared to younger adults. Older adults also exhibit altered default mode network (DMN) connectivity compared to their younger counterparts, however, the strength of connectivity was influenced by obesity status, i.e., body mass index [BMI; (Kullmann et al., 2012a)]. However, most of these studies examined group (i.e., sex, age, or obesity) differences in functional connectivity either during resting states or during cognitive performance without movement involved. Although a few studies examined functional connectivity with motor tasks, those studies were still limited to testing the movement of the small distal muscles (Karim et al., 2017) and in healthy populations, with no considerations given to group differences in the outcomes studied.

The purpose of the present study was to examine obesity and sex differences in functional connectivity changes with handgrip motor fatigue development in older adults. Functional brain activity from frontal, motor, and sensory area using functional near-infrared spectroscopy (fNIRS) were obtained from study participants as they performed isometric submaximal handgrip contractions until voluntary exhaustion. Based on a prior electroencephalogram (EEG) study of motor fatigue (Liu et al., 2007), we hypothesized that functional connectivity across the monitored areas would differ over the course of motor fatigue development. We also hypothesized that functional connectivity would differ by obesity. Finally, we explored sex differences in functional connectivity during motor fatigue.

## Materials and Methods

### Participants

Fifty-nine older adults were recruited from the local community. All participants were 65 years or older, right-hand dominant, and sedentary or recreationally active without any known musculoskeletal injuries or disorders in the past 12 months. Participants whose BMI were in the range of 18.5 ∼25 kg/m^2^ were considered as non-obese and greater than 30 kg/m^2^ were considered as obese. Six participants whose endurance times were shorter than the required duration for the functional connectivity analysis were excluded from the analysis. Table 1 lists participants’ demographics of the four experimental groups: non-obese males (*n* = 14), non-obese females (*n* = 14), obese males (*n* = 11), and obese females (*n* = 14). The Texas A&M University Institutional Review Board approved the procedures, and participants provided written informed consent before data collection.

**Table 1:** Participant demographics.

	Males			Females		
	Non-obese (*N* = 14)	Obese (*N* = 11)	*p*-value	Non-obese (*N* = 14)	Obese (*N* = 14)	*p*-value
Age	74.5 (6.4)	72.7 (6.9)	0.49	71.5 (4.2)	71.6 (5.5)	0.954
Height (cm)	177.7 (7.5)	178.4 (8.8)	0.661	163.6 (5.9)	159.9 (4.2)	0.128
Weight (kg)	76.1 (8.1)	114.8 (17.4)	<0.001	60.9 (7.0)	94.5 (14.2)	<0.001
Body mass index (kg/m^2^)	24.4 (1.2)	34.7 (3.4)	<0.001	22.9 (1.9)	37.0 (4.9)	<0.001
Waist circumference (cm)	38.9 (1.9)	50.2 (4.3)	<0.001	34.5 (3.7)	46.1 (4.1)	<0.001
Percent body fat (%)	22.8 (4.7)	35.7 (3.4)	<0.001	31.3 (5.2)	51.4 (17.4)	<0.001
Initial strength (kg)	11.2 (2.6)	12.7 (3.0)	0.198	9.5 (2.3)	8.6 (1.6)	0.252
PA (# of steps/day)	7794.8 (2259.2)	5670.7 (4791.7)	0.180	7719.7 (3104.1)	5056.9 (1782.4)	0.014

*All measures are presented as mean (SD). Statistically significant obesity differences in each sex group is tested at alpha = 0.05.*

### Procedures

**FIGURE 1:**
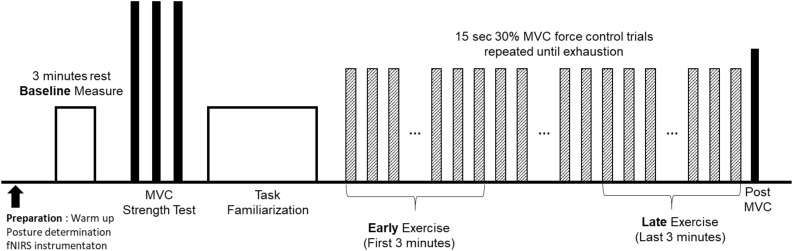
Experiment protocol for the motor fatigue experiment.

**FIGURE 2:**
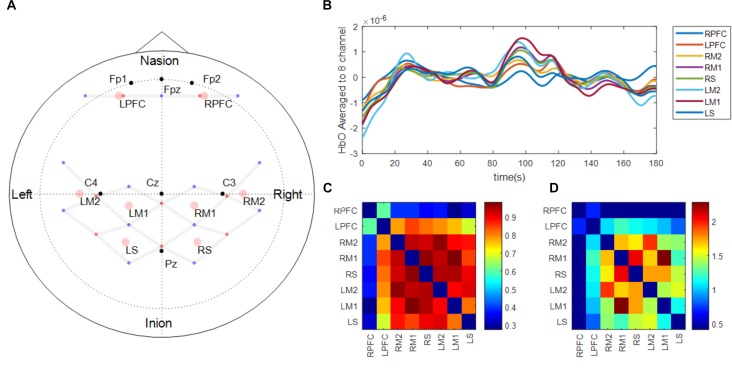
fNIRS probe design **(A)** and functional connectivity analysis procedure **(B–D)**. Panel **(A)** shows the location of light emitters (red dots) and detectors (blue dots). Gray lines between optodes depict the location of channels where brain activation is measured. Black dots represent the location of reference points based on 10–20 international EEG system. Pink circles show the location of the regions of interest which are average of surrounding channels. The regions of interest were defined based on the functions of a sensorimotor network of both hemispheres (Prefrontal area: LPFC, RPFC, Medial motor area: LM1, RM1, Lateral motor area: LM2, RM2, and Sensory area: LS, RS). Panel **(B)** shows an example of the fNIRS time-series signal processed and aggregated to the regions of interest during the early phase of one participant. Panel **(C)** shows Pearson correlation matrix across the time-series signals shown in Panel **(B)**. Panel **(D)** shows Fischer’s *z*-score converted from Panel **(C)** and only showed the scores of nodes between the two functionally connected regions based on predetermined threshold of 0.4.

The data presented in this study is the result of one experiment that is a part of a larger project that consisted of several experiment sessions. Upon informed consent, physical activity of each participant, as time-series acceleration, was measured for 1 week using three-axis accelerometer (activPAL, PAL Technologies Ltd, Glasgow, United Kingdom) attached on the right thigh. The experimenter removed the physical activity monitor on the fatigue experiment session. Data from the accelerometer provided average steps/day for each participant. Participants then attended one handgrip motor fatigue experiment, which consisted of bioinstrumentation, baseline, strength, and fatigue testings (Figure 1). Participants were first instrumented with fNIRS (Techen Inc., MA, United States, CW6 System) that measured hemodynamic responses at the frontal, motor, and sensory areas (Figure 2A). Participants sat upright with their dominant upper arm at their side, elbow flexed at 90°, and lower arm supported by an armrest. Prior to the motor fatigue task, brain activation during a baseline period was measured in which participants were asked to relax without any movement for 3 min. Following baseline tests, participants grasped a hand dynamometer (BIOPAC, CA, United States) to perform isometric handgrip contractions. After sufficient warm up (of ∼2 min), participant performed three isometric maximum voluntary contractions (MVCs) with 2 min rest in between to measure participant’s handgrip strength. The maximum value from the three MVC trials was used to determine the target force level of 30% MVC for the subsequent motor fatigue task. Participants were provided familiarization of the motor fatigue trials at 30% MVC and adequate rest before starting the fatiguing motor task. The fatigue test required participants to maintain handgrip force levels at 30% MVC for 15 s followed by 15 s rest repeatedly until voluntary exhaustion or termination. Participants were instructed to maintain their handgrip force level as closely to the target force level as possible utilizing real-time visual feedback on a computer screen. Once they terminated the fatigue task, they performed a post MVC trial.

### Cerebral Hemodynamics

Brain activation was monitored using continuous wave fNIRS (Techen Inc., MA, United States, CW6 System). The intensity of the light signal in two wavelengths (690 and 830 nm), discharged from 8 emitters and absorbed while traveling through neural tissue, were recorded at 13 detectors to obtain hemodynamic responses at a total of 26 channels. Acquired light intensities were converted into optical density by taking the logarithm of the input signal. The optical density was then low pass filtered at 3 Hz to reduce high-frequency noise. Motion artifacts that showed abrupt change were detected and corrected using spline interpolation algorithm (Scholkmann et al., 2010) and smoothed using wavelet algorithm (Chiarelli et al., 2015). Motion artifact corrected signals were bandpass filtered at 0.5 ∼0.016 Hz to reduce the effect of physiological noise and slow wave drifts. Lastly, oxygenated (ΔHbO) and deoxygenated (ΔHbR) hemoglobin at the 26 channels was calculated using modified Beer-Lambert law (Delpy et al., 1988). Location of the 21 optodes (i.e., 8 emitters and 13 detectors; Figure 2A) that constructed 26 channels where the brain activation was monitored was determined based on 10/20 international EEG system using Atlasview (Aasted et al., 2015). Since ΔHbO showed greater task related changes than ΔHbR, ΔHbO was used to analyze functional connectivity changes and task related neural activation changes during fatigue development (Malonek and Grinvald, 1996). In the present study, the ΔHbO time-series data of 26 channels were averaged into eight regions of interests (ROIs) that were defined based on the functions of a sensorimotor network of both hemispheres (Mayka et al., 2006), namely left/right prefrontal area (LPFC and RPFC, respectively), left/right medial motor area (LM1 and RM1, respectively) left/right lateral motor area (LM2 and RM2, respectively), and left/right sensory area (LS and RS, respectively). The task-related neural activation was obtained by averaging 2 s around maximum ΔHbO activation within each trial (Hyodo et al., 2016).

### Functional Connectivity Analysis

To acquire 3 min long continuous time-series signal of oxygenated hemoglobin, HbO of the first and last 12 force control trials during the motor fatigue test were extracted, concatenated, and labeled as early and late. Additionally, the 3-min baseline period was also processed and labeled as a baseline. Five participants who performed less than 24 total trials were excluded from the analysis. While concatenating HbO signal during intermittent contractions into one continuous signal, offsets between the end of the previous contraction and the beginning of the following contraction were compensated, and the concatenated signal was de-trended and low pass filtered at 0.1 Hz (Cheng et al., 2016). Pearson correlations across all ROIs were computed to determine associations between ROIs (Figures 2B,C). Pearson *R* values were then converted into Fisher’s *z*-score to determine the strength of correlations between ROIs (Figure 2D). The threshold of *z*-score to determine whether the node between the two regions are functionally connected or not was set to a predetermined value of 0.4 based on Rubinov and Sporns (2010). Based on the graph theory, each ROI in the present study was labeled as a node, and the connection between ROIs was labeled as an edge. Although the present study examined the spatial connectivity pattern between ROIs but did not perform network analysis, the terms node and edge are used subsequently (Friston, 1994). Eight nodes constructed 28 edges and the threshold to determine whether the edge between the two nodes are functionally connected were set to a predetermined value of 0.4 (Rubinov and Sporns, 2010).

### Motor Performance

Endurance time was defined as the duration between onset of the first trial and when a participant could no longer maintain the target force for more than 3 s. The rate of strength loss due to fatigue development was computed as (Initial MVC–Post MVC)/Initial MVC × 100. Handgrip forces during the strength and motor fatigue tests were recorded using the hand dynamometer (BIOPAC, CA, United States) at 1,000 Hz sampling rate. Force time-series data were low pass filtered at 15 Hz, and the middle 12 s of each 15 s force control trial was extracted. The coefficient of variation (CV = SD/average) of force was computed as a measure of force steadiness (Tracy and Enoka, 2002), and a coefficient of variation of force steadiness was computed as a measure of motor variability (Duan et al., 2018). CV of force and CV of force steadiness of first and last 12 trials were averaged and used for comparing group (obesity and sex) differences with fatigue. These variations in movement characteristics, known as motor variability, are reported to be associated with motor performance impairment and fatigability (Davids et al., 2003; Srinivasan and Mathiassen, 2012).

### Statistical Analysis

Separate paired *t*-tests between the phases; baseline vs. early, early vs. late, and baseline vs. late; of functional connectivity strength of each edge across groups pooled by sex and obesity were performed. Additionally, independent *t*-tests between obesity and sex groups, i.e., non-obese vs. obese and males vs. females, in each of the three phases (i.e., baseline, early, and late) were performed on the functional connectivity strength. The acquired *p*-values in each comparison were corrected using false discovery rate (FDR) to reduce the likelihood of false positives caused by multiple comparisons (Benjamini and Hochberg, 1995).

Separate 2 (phase: early, late) 2 (sex: male, female) × 2 (obesity: non-obese, obese) × 2 (side: left, right) mixed factor ANOVAs were conducted on ΔHbO values from each of the four ROIs (prefrontal, medial motor, lateral motor, and sensory area). Statistical significances of each ROI were corrected using FDR (Benjamini and Hochberg, 1995). Handgrip strength loss after fatigue development and endurance times were submitted to separate 2 (sex: male, female) × 2 (obesity: non-obese, obese) factorial ANOVAs to examine sex and obesity differences on handgrip motor fatigue. Additionally, CV of force (force steadiness) and CV of CV were submitted to a 2 (phase: early, late) × 2 (sex: male, female) × 2 (obesity: non-obese, obese) ANOVA to examine group (obesity and sex) and phase differences on motor performance during the fatigue test. Statistical significance was testing with alpha = 0.05, and *post hocs* were conducted as needed using Bonferroni corrections.

## Results

### Functional Connectivity

**FIGURE 3:**
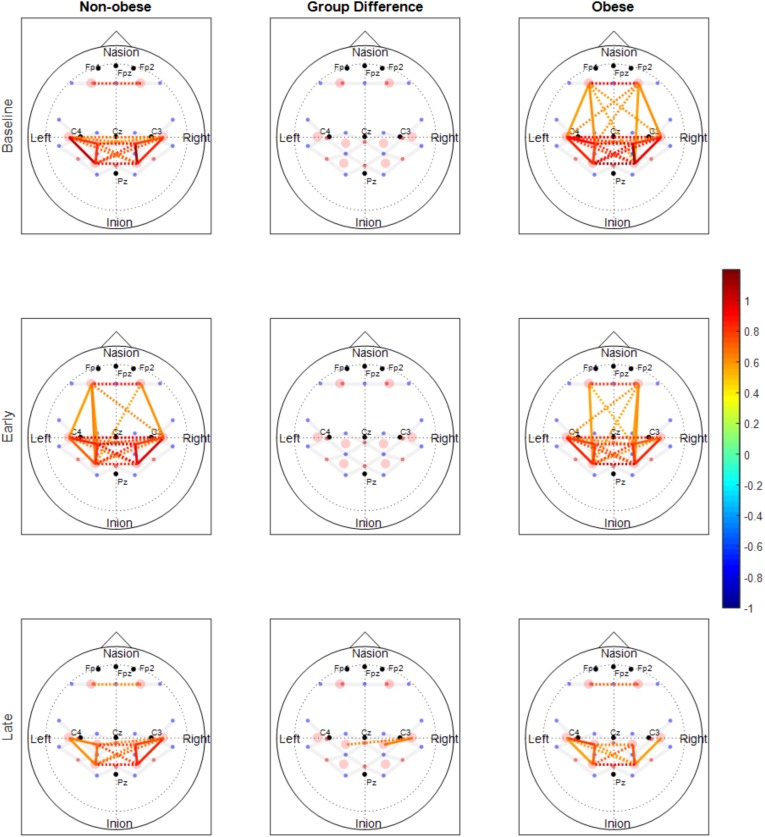
Functional connectivity maps of non-obese (left column) and obese (right column) during the baseline and motor fatigue test. The connectivity maps are produced from the weighted matrix, and the color of each node depicts the strength of connectivity based on provided color scale. Nodes with solid lines indicate intra-hemispheric connectivity, and nodes with dotted lines indicate inter-hemispheric connectivity. Middle column shows the nodes which connectivity was significantly different between the obesity groups. The connectivity difference maps are produced from the binary matrix, and positive score based on the provided color scale indicates connectivity of the nodes were significantly stronger for non-obese than obese.

**FIGURE 4:**
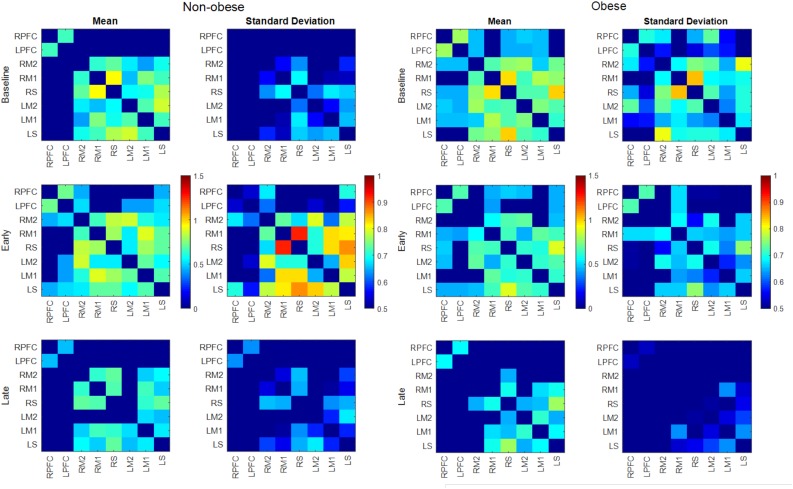
Mean and SD of connectivity matrix for each phase in the non-obese (Left) and obese (Right) groups. Color matrices are produced out of Fischer’s *Z*-score.

**FIGURE 5:**
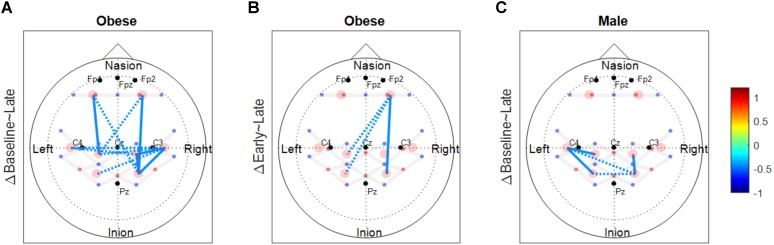
Functional connectivity changes between phases. These connectivity change maps are produced from the binary matrix, and the scores based on the provided color scale indicates either the connectivity was significantly decreased (negative score) or not. **(A)** Significantly decreased connectivity between baseline and late phases in the obese group. **(B)** Significantly decreased connectivity between early and late phases in the obese group. **(C)** Significantly decreased connectivity between baseline and late phases in males. Solid lines indicate significant connectivity changes within each hemisphere and dotted lines indicate significant connectivity changes between the two hemispheres.

The number of functionally connected edges decreased during the late phase (non-obese: 13, obese: 11) compared with the baseline phase (non-obese: 16, obese: 24) and the early phase (non-obese: 22, obese: 21). Spatial connectivity patterns were different during the baseline phase but the differences between the non-obese and obese groups were not significant (Figure 3). However, the obese group showed greater variability in connectivity (Figure 4). Motor task related spatial connectivity patterns between non-obese and obese groups were observed to be similar during the early phase (Figure 3), however, the variability of connectivity was found to be increased for non-obese group (Figure 4). During the late phase, the non-obese group demonstrated significantly greater connectivity between right lateral motor area and medial motor area of both hemispheres when compared to the obese group. The obese group showed most distinctive fatigue-related functional connectivity changes and the connectivity during the late phase was lower than during the baseline and early phases (Figures 5A,B).

**FIGURE 6:**
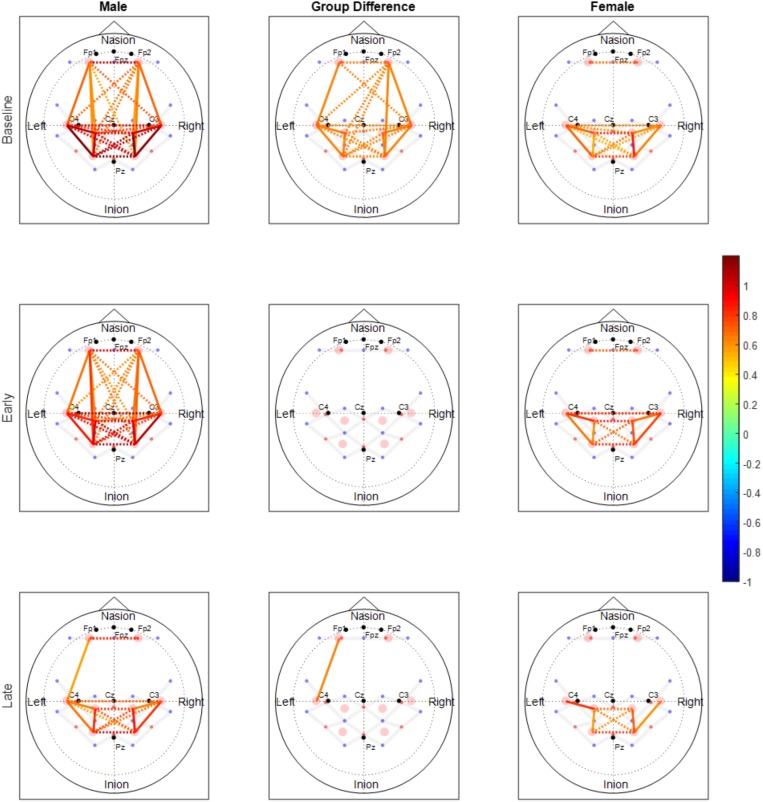
Functional connectivity maps of the male (left column) and female (right column) groups during the baseline and motor fatigue test. The connectivity maps are produced from the weighted matrix, and the color of each node depicts the strength of connectivity based on provided color scale. Nodes with solid lines indicate intra-hemispheric connectivity, and nodes with dotted lines indicate inter-hemispheric connectivity. Middle column shows the nodes where connectivity was significantly different between the sexes. The connectivity difference maps are produced from the binary matrix, and positive score based on the provided color scale indicates connectivity of the nodes were significantly stronger for males than for females.

**FIGURE 7:**
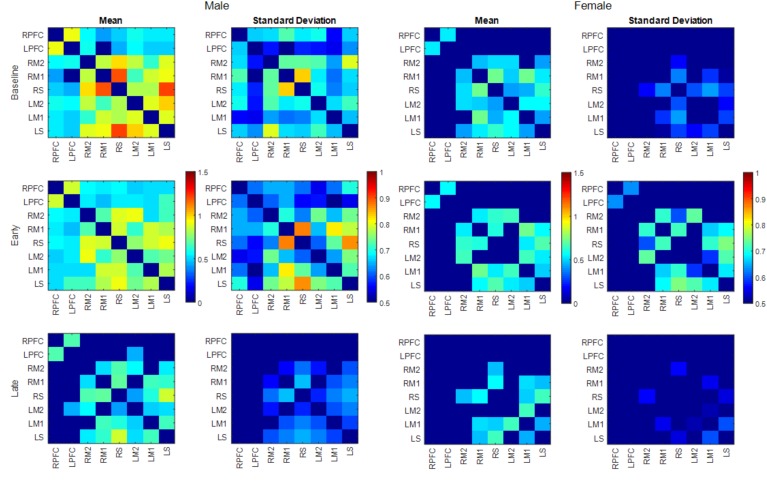
Mean and SD of connectivity matrix for each phase in males (left) and females (right). Color matrices are produced out of Fischer’s *Z*-score.

Both males and females exhibited fewer functionally connected edges during the late phase (males: 15, females: 8) when compared with the baseline (males: 27, females: 15) and the early phases (males: 28, females: 12). During the baseline and early phases, males exhibited functional connectivity that was greater than the threshold at 27 edges across all nodes but females showed connectivity within the prefrontal and sensorimotor areas, however, the two areas were not functionally connected (Figure 6). Functional connectivity changes between the baseline and early phases were not found to be significant. While the sex differences in connectivity were observed to exist at all three phases (as seen in Figures 6, 7), connectivity between the two groups was not significantly different due to the large variability observed during the early phase (Figure 7). Both groups showed decreased connectivity and decreased the number of connected edges with motor fatigue development. During the late phase, males showed significantly stronger connectivity at a node between left prefrontal area and left lateral motor area. Also, males showed decreased connectivity with motor fatigue during the late phase compared to the baseline phase at the sensorimotor area (Figure 5C).

### Changes in Cerebral Hemodynamics

**FIGURE 8:**
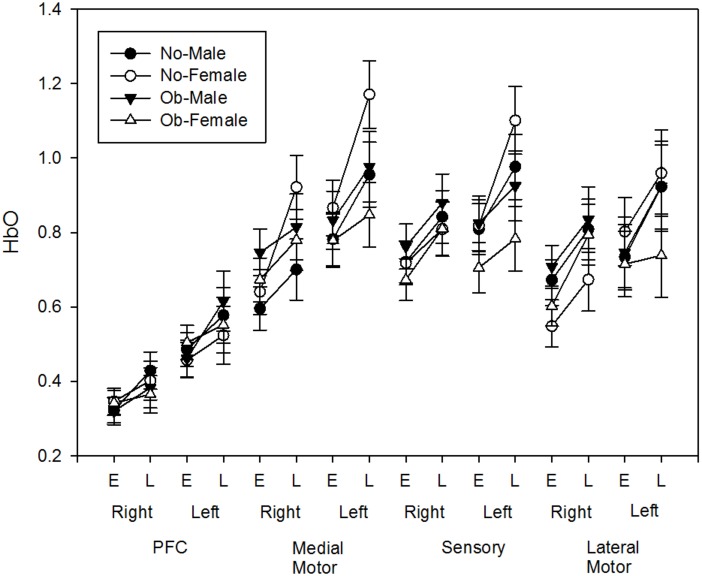
Fatigue-related HbO changes (Early and Late) at each region of interest (PFC, Medial Motor, Sensory, Lateral Motor) of both hemisphere (Left, Right).

Task related ΔHbO increased with fatigue development [*F*(1,49) = 26.627, *p* < 0.001] at all channels and strong contralateral activation was observed at all ROI after FDR correction (all *p*-values <0.005, Figure 8). No obesity or sex differences were found (all *p*’s > 0.4). However, a side × obesity × phase interaction was found in sensory motor area [*F*(1,49) = 9.821, *p* = 0.003]. *Post hoc* revealed that the fatigue-related increase in ΔHbO in the contralateral sensory area was greater for non-obese adults when compared to obese adults.

### Changes in Motor Performance

Table 2 lists the means (SD) of the motor performance data across the obesity and sex groups. Fatigue-related strength loss did not differ by obesity (*p* = 0.347), or sex (*p* = 0.759), or their interaction (*p* = 0.255). Similarly, endurance time did not differ by obesity (*p* = 0.595), or sex (*p* = 0.859), or their interaction (*p* = 0.943). CV of force during motor fatigue increased during the late phase [*F*(1,48) = 17.436, *p* < 0.001] when compared to the early phase, however, no group differences, or their interactions with each other or with phase were found (both *p*’s > 0.16). CV of CV decreased during the late phase when compared to the early phase [*F*(1,46) = 21.219, *p* < 0.001], in males than females [*F*(1,48) = 17.193, *p* < 0.001], and in obese adults than in non-obese adults [*F*(1,48) = 3.963, *p* = 0.052]. Additionally, an obesity × phase interaction was found on CV of CV [*F*(1,46) = 3.875, *p* = 0.055], with the non-obese group exhibiting decreased CV of CV as they fatigued, but the obese adults exhibiting reduced CV of CV across both phases of fatigue.

**Table 2:** Motor fatigue and performance measures are presented as mean (SD).

		Male		Female	
		Normal (*N* = 14)	Obese (*N* = 11)	Normal (*N* = 14)	Obese (*N* = 14)
Strength loss (%)		37.5 (10.6)	32.1 (6.9)	33.4 (10.2)	35.4 (8.2)
Endurance time (s)		1287.9 (513.5)	1217.5 (376.3)	1272.7 (629.0)	1187.1 (493.9)
CV force (%)	Early	1.8 (0.6)	1.9 (0.3)	1.8 (0.5)	2.1 (0.6)
	Late	2.7 (2.5)	2.1 (0.3)	1.9 (0.6)	2.4 (0.7)
CV force steadiness (%)	Early	19.6 (4.4)	15.3 (6.1)	28.8 (11.4)	22.6 (9.2)
	Late	16.2 (4.1)	12.2 (4.6)	18.2 (7.5)	18.9 (5.8)

## Discussion

### Obesity Differences in Functional Connectivity

During the baseline phase, the obese group showed more functionally connected edges across most nodes while non-obese group only showed interhemispheric connectivity within each prefrontal and sensorimotor area, but not between the two areas. Previous studies reported obesity-related differences in functional networks such as, altered DMN (Kullmann et al., 2012a; García-García et al., 2013a) and increased connectivity within salience network (Kullmann et al., 2012b; García-García et al., 2013b). However, this obesity effect is limited to the differences in the functional networks. The connectivity patterns observed in the present study may indicate overall greater connectivity of the obese group during the baseline phase, but the use of pooled datasets across sexes in this study hinders isolating the said effect.

The number of functionally connected nodes during early phase increased for the non-obese group but decreased with the obese group. Both groups showed connectivity patterns in which edges of both areas and hemispheres were connected (Figure 3). Given that different spatial connectivity pattern between the sexes was maintained during the motor task, relatively similar spatial connectivity pattern during early phase indicates the motor task influences differently when compared across the sex or obesity groups. These differences in connectivity change patterns are in agreement with the studies reported sex dependent structural connectivity (Tomasi and Volkow, 2012; Ingalhalikar et al., 2014) and obesity dependent functional connectivity (Kullmann et al., 2012b; García-García et al., 2013a,b). During the late phase, the number of functionally connected edges and variability of connectivity decreased (Figures 3, 4).

### Sex Differences in Functional Connectivity

The spatial patterns of functionally connected edges, in which connectivity scores were greater than the threshold value, were different between males and females (Figure 6). While males showed connectivity between the frontal and sensorimotor areas and between the hemispheres, females showed only inter-hemispheric connectivity within each area. This is partially in congruence with the study that reported stronger intra hemispheric connectivity for males and stronger inter-hemispheric connectivity for females (Ingalhalikar et al., 2014). During the baseline phase, which can be considered as a resting state when compared with other connectivity studies, males showed stronger connectivity with both inter/intra network connectivity that suggests sex related different localized brain activation pattern (Tomasi and Volkow, 2012). In addition, males exhibit greater variability in connectivity than females which is congruent with the result reported greater functional activations for males while females had greater structural connectivity densities (Keller and Menon, 2009).

The spatial pattern of functionally connected edges of both sexes did not change from that during the baseline phase and the connectivity pattern at the frontal and sensorimotor area is consistent with that Yao et al. (2016) reported. However, the group difference observed during baseline phase was negated (Figure 6) due to overall greater variability in both sexes (Figure 7). These results suggest that the handgrip motor task did not significantly alter the spatial functional brain network. While older adults tend to over-recruit neural area to perform motor tasks compared with young adults (Mattay et al., 2002; Heuninckx et al., 2008), both contralateral and ipsilateral functional connectivity observed in the present study is consistent with concurrent increase of hemodynamic response with motor tasks in both contralateral and ipsilateral hemispheres, and variability in connectivity at the node that was acquired from the averaged brain activation of surrounding ROIs is indicative of sex and task dependent different changes in neural recruitment in older adults. During the late phase, the number of functionally connected edges was decreased as well as the variability of connectivity in both sexes. This result suggests a dissociation of the brain activation pattern among the areas that used to be associated with fatigue development.

### Functional Connectivity Changes During Motor Fatigue Development

Motor fatigue development was associated with changes in functional connectivity in older adults, across all groups. Older adults, regardless of sex and obesity differences, exhibited fewer functionally connected edges as fatigue developed, and the lower variability of the connectivity patterns (pooled across all participants) observed implied that the decline in connectivity was homogenous across all groups (Figures 3, 6). Only a few studies examined functional connectivity associated with acute motor fatigue and these studies employed EEG or fMRI (Liu et al., 2007). For example, Peltier et al. (2005) reported decreases in interhemispheric connectivity in the motor areas after fatigue. The findings from the present study, which demonstrated decreased interhemispheric connectivity with fatigue development [particularly in the obese groups (Figures 5A,B) and male (Figure 5C)] are in agreement with Peltier et al. (2005). However, the present study examined connectivity during fatigue development whereas the previous study measured resting state functional connectivity after motor task.

### Force Variability and Brain Activity Changes During Motor Fatigue Development

In the present study, similar to previous studies examining force variability patterns with fatigue (Srinivasan and Mathiassen, 2012), fatigue development here was associated with decreased force steadiness and lower variability of this reduced motor performance, i.e., a decrease in CV of force steadiness. There were no obesity or sex differences in endurance time, strength loss, or force steadiness. Endurance and strength loss have shown to differ with obesity and sex, however, in older adults, the differences are largely observed in postural and/or lower extremity muscles (Hunter, 2016, 2017). Understanding force variability patterns can provide a greater resolution on how individuals differently adapt or correct for motor demands (Mathiassen et al., 2003; Madeleine, 2010). In the present study, lower variability of CV was found for males than females. Given that both males and females showed similar steadiness patterns over time, increased variability in the patterns exhibited by females may be attributed to different neural adaptation strategies than males to maintain motor performance (Hunter, 2014; Duan et al., 2018). This was associated with concomitant sex differences in the functional connectivity patterns observed in the present study.

Similar to males, the obese group exhibited lower variability of force steadiness than the non-obese group, which was accompanied by fatigue-related concomitant declines in the frontal-motor connectivity and reduced activation in sensory motor areas with obesity. Obesity has shown to adversely impact brain structure and function, particularly of the frontal lobes (Gustafson et al., 2004; Pannacciulli et al., 2006; Whitmer et al., 2008). We have previously reported associations between impaired functioning of the frontal lobe and obesity-related decrements in upper extremity motor performance (Mehta and Shortz, 2014) and gait speed (Osofundiya et al., 2016). Thus, it is likely that obesity may contribute to reduced cortical excitation of frontal-motor areas in older adults, which may impact functional motor outcomes in this population. It is likely that physical activity of the study participants may have confounded obesity differences in the study variables. While PA was similar between non-obese and obese males, obese females exhibited lower steps/day than non-obese females. Moreover, the obesity groups were significantly different based on BMI, percent body fat, and waist circumference values. Given that none of the study variables were affected by obesity × sex interactions, it is likely that the differences observed here are largely attributed to obesity-related changes in neural and muscular mechanisms.

### Study Limitations

Due to the limited spatial resolution and measurement depth of fNIRS, connectivity across the various functional networks in the whole brain was not available to measure. Instead, we measured the brain activation of an inch depth of the targeted cortical area of frontal and sensorimotor area. This limitation can potentially be resolved with multi-modal neuroimaging such as fNIRS combined with EEG or fMRI. Second, although the sex and obesity groups formed four groups, we used pooled datasets for functional connectivity analysis due to a relatively small sample within each of the four groups. However, we tested obesity and sex interactions with functional brain activation and motor performance measures. Finally, PA levels of the study participants may potentially confound findings. Future work is warranted that addresses these limitations and examine possible relationships between the force variability and functional connectivity patterns.

## Conclusion

The present study examined changes in functional connectivity patterns during handgrip motor fatigue in older adults. We measured brain activity at the frontal and sensorimotor area using fNIRS, and computed and compared functional connectivity across obesity and sex groups. Motor fatigue-related functional connectivity changes over time was found along with fatigue-related motor performance changes as decreases in force production and force steadiness as well as increases in contralateral frontal and sensorimotor activation. We also found both obesity- and sex-specific functional architecture and activation differences during motor fatigue in older adults, and that these differences were accompanied by obesity and sex differences in variability of force steadiness implicating group-specific neural adaptation strategies to maintain overall fatigue outcomes (i.e., similar endurance times and strength loss). Findings reported here may facilitate development of targeted interventions to offset motor impairments in vulnerable older adults.

## Ethics Statement

This study was carried out in accordance with the recommendations of the Federal Regulations for Protection of Human Research Subjects (45 CFR 46) with written informed consent from all subjects. All subjects gave written informed consent in accordance with the Declaration of Helsinki. The protocol was approved by the Institutional Review Board at Texas A&M University.

## Author Contributions

RM conceptualized and designed the study. JR collected, processed, and analyzed data. RM and JR interpreted the results and wrote the manuscript.

## Conflict of Interest Statement

The authors declare that the research was conducted in the absence of any commercial or financial relationships that could be construed as a potential conflict of interest.
